# Recurrent adenylation domain replacement in the microcystin synthetase gene cluster

**DOI:** 10.1186/1471-2148-7-183

**Published:** 2007-10-01

**Authors:** David P Fewer, Leo Rouhiainen, Jouni Jokela, Matti Wahlsten, Kati Laakso, Hao Wang, Kaarina Sivonen

**Affiliations:** 1 Department of Applied Chemistry and Microbiology, PO Box 56, Viikki Biocenter, Viikinkaari 9, FIN-00014, University of Helsinki, Finland; 2 Valio Ltd, R&D, PO Box 30, FIN-00039 Valio, Helsinki, Finland

## Abstract

**Background:**

Microcystins are small cyclic heptapeptide toxins produced by a range of distantly related cyanobacteria. Microcystins are synthesized on large NRPS-PKS enzyme complexes. Many structural variants of microcystins are produced simulatenously. A recombination event between the first module of *mcyB (mcyB1) *and *mcyC *in the microcystin synthetase gene cluster is linked to the simultaneous production of microcystin variants in strains of the genus *Microcystis*.

**Results:**

Here we undertook a phylogenetic study to investigate the order and timing of recombination between the *mcyB1 *and *mcyC *genes in a diverse selection of microcystin producing cyanobacteria. Our results provide support for complex evolutionary processes taking place at the *mcyB1 *and *mcyC *adenylation domains which recognize and activate the amino acids found at X and Z positions. We find evidence for recent recombination between *mcyB1 *and *mcyC *in strains of the genera *Anabaena*, *Microcystis*, and *Hapalosiphon*. We also find clear evidence for independent adenylation domain conversion of *mcyB1 *by unrelated peptide synthetase modules in strains of the genera *Nostoc *and *Microcystis*. The recombination events replace only the adenylation domain in each case and the condensation domains of *mcyB1 *and *mcyC *are not transferred together with the adenylation domain. Our findings demonstrate that the *mcyB1 *and *mcyC *adenylation domains are recombination hotspots in the microcystin synthetase gene cluster.

**Conclusion:**

Recombination is thought to be one of the main mechanisms driving the diversification of NRPSs. However, there is very little information on how recombination takes place in nature. This study demonstrates that functional peptide synthetases are created in nature through transfer of adenylation domains without the concomitant transfer of condensation domains.

## Background

Planktonic cyanobacteria often form heavy scums or blooms in freshwater lakes, ponds and reservoirs worldwide [1]. Cyanobacterial blooms constitute a health-risk for human beings via recreational or drinking water through the production of a range of hepatotoxins and neurotoxins [1]. Microcystins are a diverse group of low molecular weight cyclic heptapeptides and are the most common hepatotoxins produced by cyanobacteria. They are potent inhibitors of eukaryotic protein phosphatases 1 and 2A [2] and are linked to the deaths of wild animals and livestock worldwide [1].

**Figure 1:**
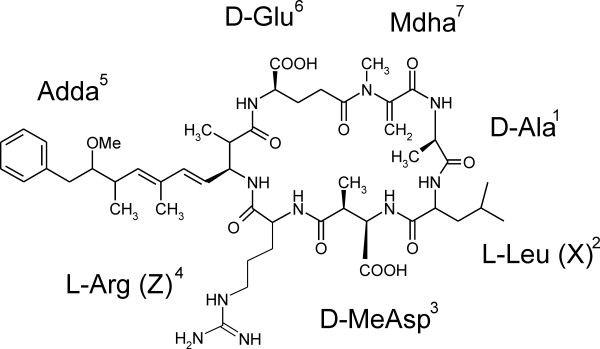
**The highly toxic microcystin-LR variant**. The microcystin chemical structure can be generalized as cyclo-D-Ala^1^-X^2^-D-MeAsp^3^-Z^4^-Adda^5^-D-Glu^6^-Mdha^7 ^where X and Z denote the highly variable second and fourth positions. Microcystins may contain L-Ala, L-Arg, L-Glu, L-Hil, L-Hph, L-Hty, L-Leu, L-Met, L-Phe, L-Try, L-Tyr, or L-Val at the X position and L-Aba, L-Ala, L-Arg, L-Glu, L-Har, L-Leu, L-Met, L-Phe, L-Try, or L-Tyr at the Z position [1].

There are over 65 structural variants of microcystins differing in modifications to the peptide backbone or the type of amino acids incorporated into the microcystin [1]. The general structure of microcystins can be summarized as cyclo-D-Ala^1^-X^2^-D-MeAsp^3^-Z^4^-Adda^5^-D-Glu^6^-Mdha^7^where X and Z are variable L-amino acids (Figure 1). Many of these microcystin variants are synthesized simultaneously by the producing cyanobacterium [1]. Structural variation has been encountered at all seven positions, but the highest degree of structural variation is found at the X and Z positions (Figure 1). The two most common microcystin variants, microcystin-LR and microcystin-RR, contain L-Leu or L-Arg at the X position and L-Arg at the Z position in the final cyclic heptapeptide. However, microcystins may also contain other proteinogenic, non-proteinogenic and dicarboxylic acids at these positions [1]. Structural variants of microcystin do not have the same toxicities and microcystin-LR is an order of magnitude more toxic than microcystin-RR [1].

Microcystins are mainly produced by planktonic strains of the distantly related cyanobacterial genera *Anabaena, Microcystis *and *Planktothrix *[1]. Microcystin production is also known from a small number of planktonic, benthic and terrestrial strains of the genera *Nostoc *[3-5], *Hapalosiphon *[6], and *Phormidium *[7]. Insertional mutagenesis has demonstrated that all microcystin variants produced by *Microcystis aeruginosa *S-70, K-139 and PCC 7806 are synthesized by an enzyme complex encoded in a single 55-kb gene cluster [8-10]. The enzyme complex which directs the biosynthesis of microcystins includes peptide synthetases, polyketide synthases, mixed peptide synthetases-polyketide synthases, and tailoring enzymes [9-13]. Phylogenetic analyses suggest that the microcystin synthetase gene cluster was present in the last common ancestor of all present-day producer organisms [14]. The sporadic distribution of microcystin synthetase gene clusters among cyanobacteria is proposed to be the result of gene loss rather than recent horizontal gene transfer [14,15].

Many important antibiotics, siderophores and toxins are synthesized on NRPS enzyme complexes [16]. NRPSs possess a highly conserved modular structure with each module being comprised of catalytic domains responsible for the adenylation, thioester formation and in most cases condensation of specific amino acids [16]. The arrangement of these domains within the multifunctional enzymes determines the number and order of the amino acid constituents of the peptide product [17]. Additional domains for the modification of amino acid residues such as epimerization, heterocyclisation, oxidation, formylation, reduction or *N*-methylation may also be included in the module [16-18]. The modular structure of NRPSs allows the rational design of novel peptides by targeted replacement of these catalytic domains [19]. The adenylation domain appears to be the primary determinant of substrate selectivity in NRPSs [[17] and others]. High structural conservation of the adenylation domain allows prediction of amino acids lining the putative binding pocket which determines substrate specificity [20]. However, recent studies predict an editing function for the condensation domain suggesting that condensation and adenylation domains in artificial junctions may be incompatible and block peptide synthesis [17]. This finding lead to the hypothesis that in nature condensation and adenylation domains may act as an inseparable couple and be transferred together during natural rearrangements of NRPS gene clusters [18,21].

**Figure 2:**
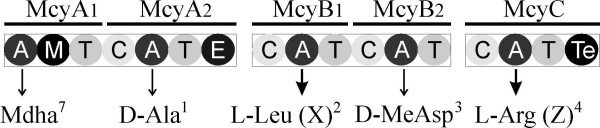
**The modular organization of McyA, McyB and McyC proteins**. These three proteins catalyze 6 rounds of elongation and the final cyclisation of the heptapeptidyl microcystin intermediate. The McyB1 and McyC adenylation domains are responsible for the recognition and activation of the amino acids found at the X and Z positions in the microcystin respectively [11]. The McyB1 and McyC condensation domains are responsible for peptide bond formation between this activated amino acid and the growing peptide chain [11]. Each circle represents a NRPS enzymatic domain: A, aminoacyl adenylation; M, *N*-methyltransferase; T, Thiolation domain, C, condensation; E, epimerization; Te, thioesterase.

The amino acids incorporated at the X and Z positions in structural variants of microcystin are recognized and activated by the McyB1 and McyC adenylation domains (Figure 2). Recombination between the adenylation domains of *mcyB1 *and *mcyC *is linked to the production of microcystin-RR by strains of the genus *Microcystis *[22]. Recombination is thought to be an important factor contributing to the genetic diversity of the microcystin synthetase gene cluster in strains of the genus *Microcystis *[23]. However, it is not clear how widespread this phenomenon is in other microcystin producers or if the condensation domain is also transferred with the adenylation domain. Here we undertake a multigene phylogenetic study in order to investigate the number and timing of recombination events during the evolution of the microcystin synthetase gene cluster in a variety of microcystin producing cyanobacteria. We show clear evidence for the recurrent exchange and replacement of the adenylation domain without the concomitant transfer of the condensation domain in a broad range of microcystin producing cyanobacteria.

## Results

### Structural characterization of the identified microcystins

**Figure 3:**
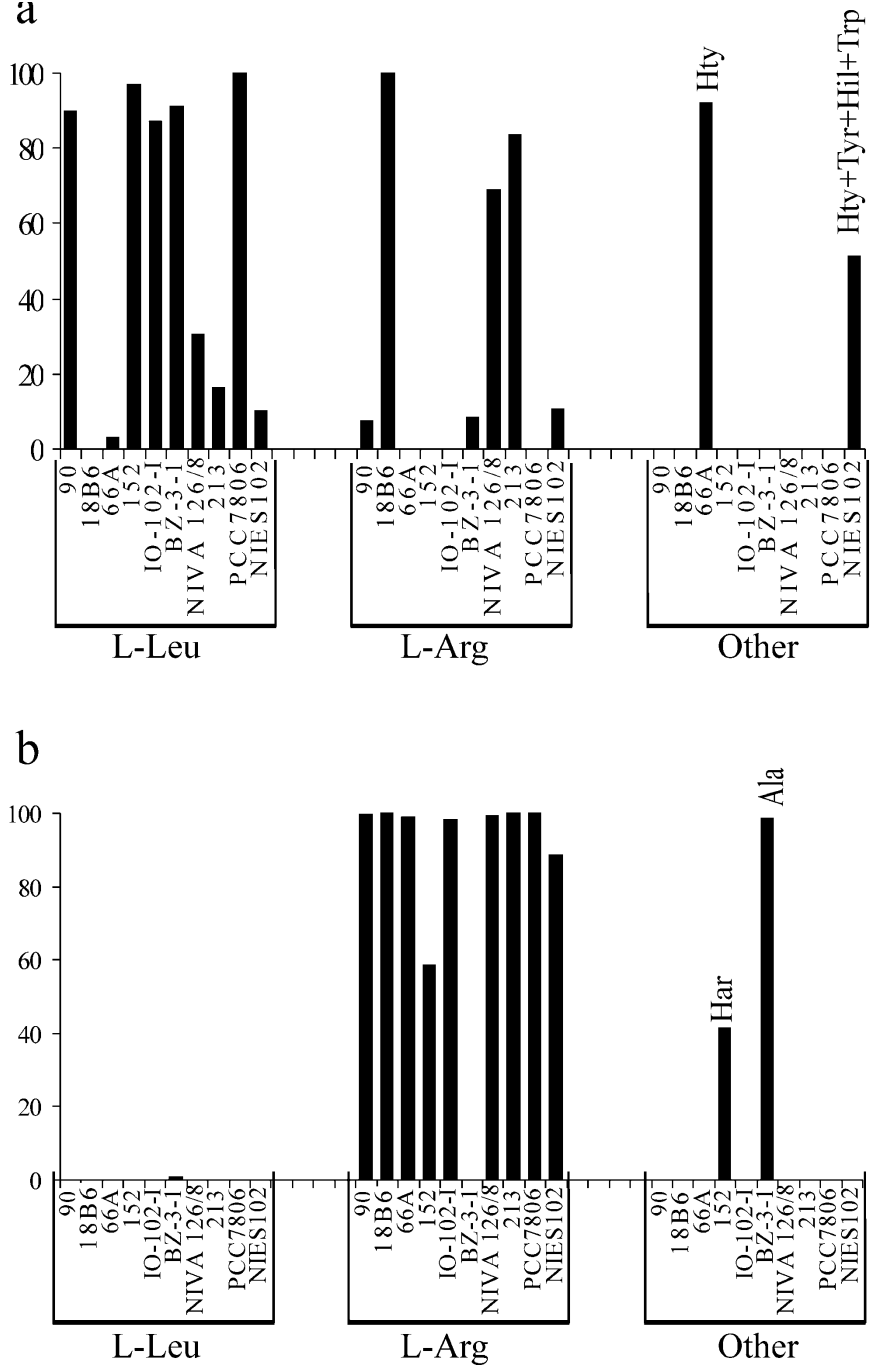
**The relative proportions of amino acids incorporated into the X and Z position**. The relative proportions of amino acids incorporated into the X and Z position of the microcystins produced by the strains included in this study as determined by LC-MS. *(a) *The amino acids present at the X position in microcystins. These amino acids are recognized and activated by the McyB1 adenylation domain. *(b) *The amino acids present at the Z position in microcystins. These amino acids are recognized and activated by the McyC adenylation domain. The structures and percentages of individual microcystins produced by the 10 strains of cyanobacteria included in this study are given in the supplementary information section (see additional file 1).

We documented the simultaneous production of 3 to 47 microcystin variants in these strains (see additional file 1). The microcystin variants produced by these strains differed in the methylation of the α-amino group of Mdha, the β-carboxyl of D-MeAsp and the C9 hydroxyl of Adda. However, most structural differences lay in the type of amino acid incorporated at the X position. Most strains produced microcystins that contained L-Leu at the X position (Figure 3a). The strains included in this study also produced microcystins which contained L-Arg, L-Hil, L-Hph, L-Hty, L-Phe, L-Try, L-Tyr, or L-Val at the X position (see additional file 1). Most strains produced microcystins that contained L-Arg at the Z position (Figure 3b). However, almost half of the microcystin variants contained L-Har at the Z-position in *Nostoc *sp. 152 (Figure 3b). Almost all variants produced in *Hapalosiphon hibernicus *BZ-3-1 contained L-Ala at the Z-position but this strain also produced minor microcystins variants in trace amounts that contained L-Leu or L-Val at this position (see additional file 1). The strains included in this study produced a wide range of common and rare microcystins. A large number of minor microcystin variants were identified through characteristic UV spectra. However, in most cases the low amounts of microcystins produced prevented characterization of the total structures.

### Recombination breakpoints in mcyB1 and mcyC

**Figure 4:**
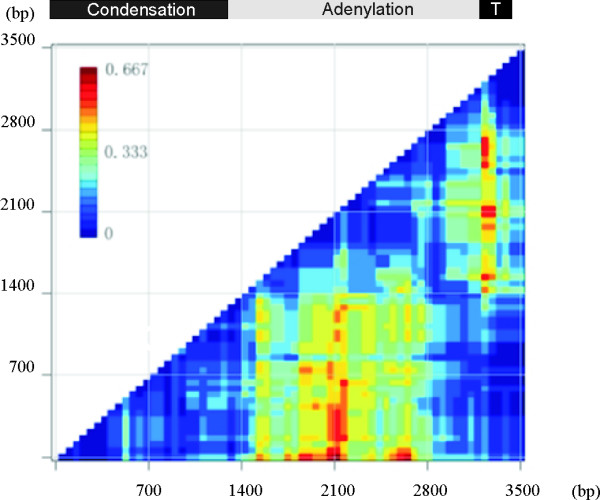
**A phylogenetic compatibility matrix of *mcyB1 *and *mcyC *genes from 10 strains of toxic cyanobacteria**. A phylogenetic compatibility matrix of *mcyB1 *and *mcyC *genes from 10 strains of toxic cyanobacteria. The matrix was constructed through comparing congruence between subtrees of whole alignment. At first, 67 alignment fragments were obtained by moving a 300 nucleotide window along the alignment with a step of 50 bases, and neighbor-joining tree of each fragment was constructed by PHYLIP. Then phylogenetic violations of any two different subtrees were calculated by TREEORDERSCAN (Simmonic 2005 version 1.5), and proportionally presented as a colour gradient showed in the figure. The NRPS enzymatic domains present in McyB1 and McyC are indicated: A, aminoacyl adenylation; C, condensation; T, Thiolation domain; Te, Thioesterase.

**Figure 5:**
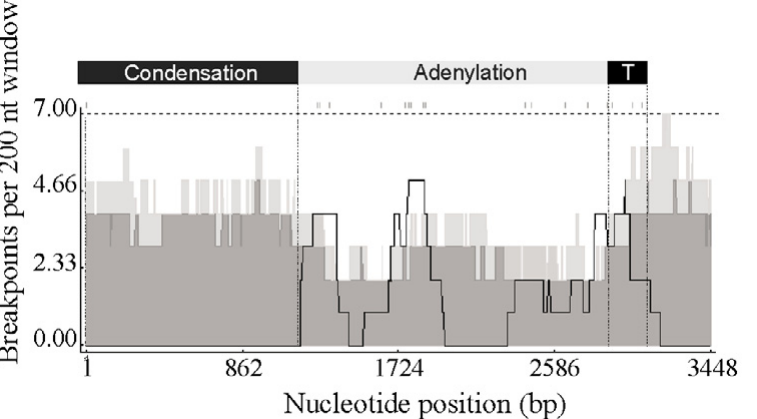
**Breakpoints density plot along the alignment of *mcyB *and *mcyC *genes**. Light grey and dark grey areas respectively indicate local 99% and 95% breakpoint clustering thresholds taking into account regional differences in sequence diversity that influence the ability of different methods to detect recombination breakpoints. The broken line in the breakpoint density graph indicate 95% confidence thresholds for globally significant breakpoint clusters. The boundary between the condensation, adenylation and thiolation domains is indicated with a solid line.

Phylogenetic-compatibility analysis indicated extensive incongruence between the adenylation and condensation domains of *mcyB1 *and *mcyC *(Figure 4). Analysis of the nucleotide sequences of *mcyB1 *and *mcyC *identified recombination breakpoints in the adenylation and thiolation domains using six different methods to detect recombination (Figure 5). The recombination area extended across the entire adenylation domains spanning conserved core motifs A1–10 into the middle of the thiolation domain (Figure 5). Additional sets of breakpoints were identified within the adenylation domain in *Anabaena *sp. 18B6 replacing the adenylation domain elements A2–A10 (data not shown). In the case of *Microcystis aeruginosa *PCC7806 a second set of breakpoints were also identified spanning the substrate conferring portions of the adenylation domain between the core motifs A3–A8 (data not shown).

### Phylogenetic analysis of McyB1 and McyC condensation and adenylation domains

**Figure 6:**
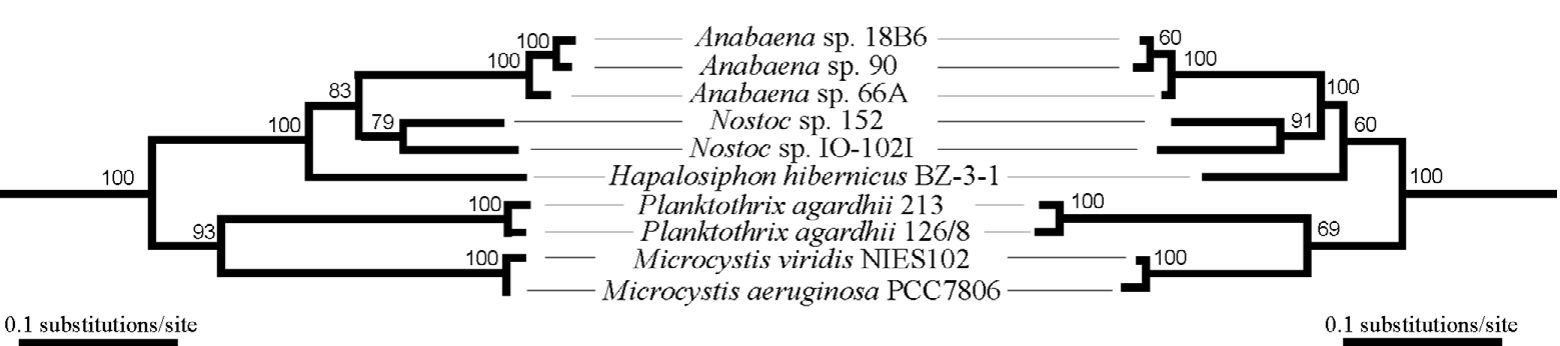
**Phylogenetic congruence between housekeeping and microcystin synthetase genes**. Congruence between housekeeping genes of the producer organism (16S rRNA, *rpoC1*, *rpoB, tufA *and *rbcL*) on the left and the microcystin synthetase genes (*mcyD, mcyE*, and *mcyG*) on the right. Maximum-likelihood tree based on five housekeeping genes (-lnL = 20872.57747) and 3 microcystin synthetase genes (-lnL 21445.80119). Bootstrap values above 50% from 1000 maximum-likelihood bootstrap replicates are given at the nodes. Branch lengths are proportional to sequence.

**Figure 7:**
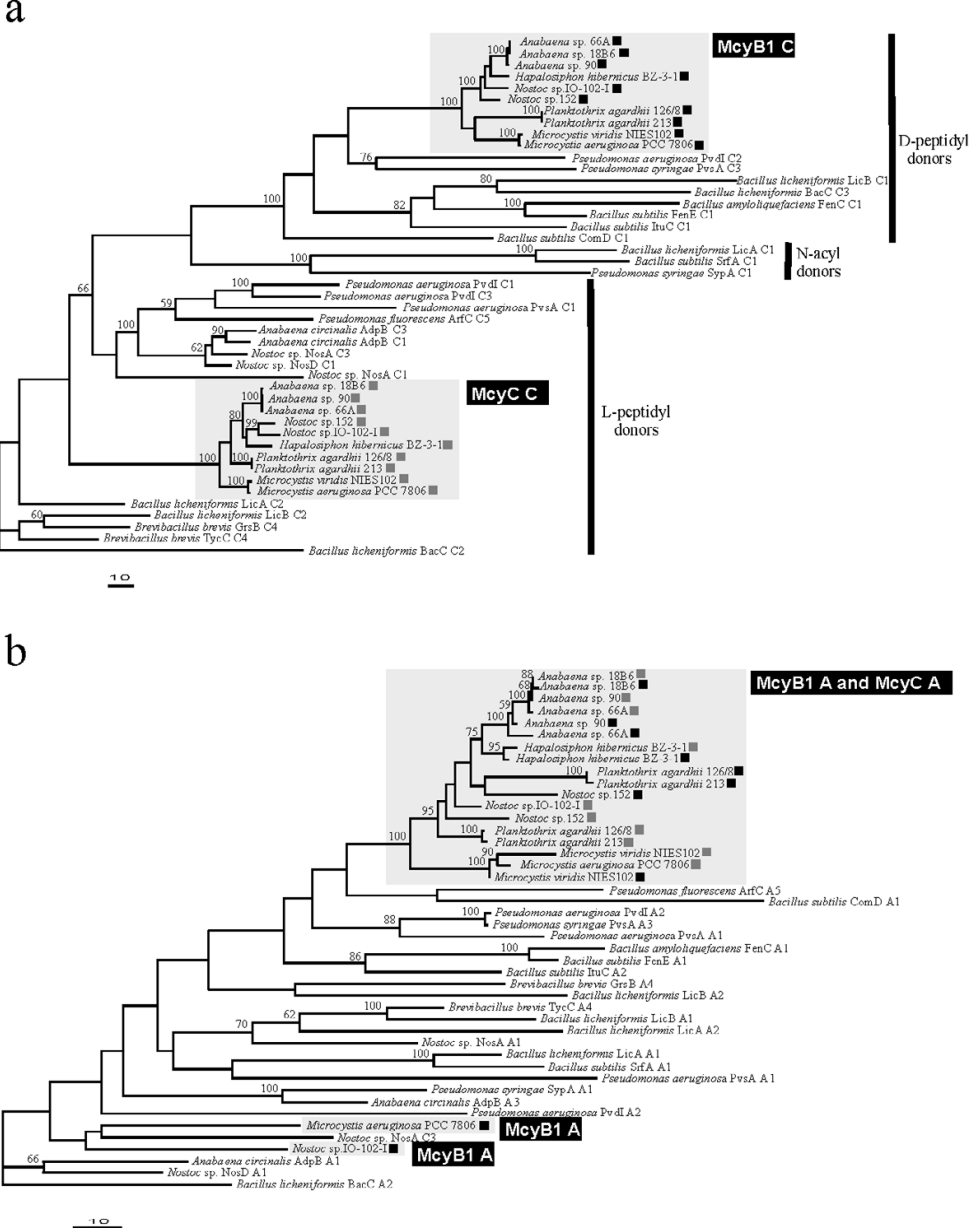
**Discordant phylogenetic relationships between the McyB1 and McyC condensation and adenylation domains**. *(a) *A maximum-likelihood tree based on the McyB1 and McyC condensation domains (C) reflecting separate evolutionary history for these two condensation domains indicating the chirality of the amino acid at the donor site of the condensation domain (-lnL = 21740.48206). *(b) *A maximum-likelihood tree based on the McyB1 and McyC adenylation domains (A), from A3–A8, showing intermixed cluster of McyB1 and McyC adenylation reflecting a more recent evolutionary history characterized by periods of replacement through recombination leading to domain replacement (-lnL = 10308.24462).

Maximum-likelihood trees based over 7,000 bp of nucleotide data from 5 housekeeping genes and 3 microcystin synthetase genes were congruent and each topology received robust bootstrap support (Figure 6). Maximum-likelihood trees based on the amino acid sequence of the condensation domain from McyB1 and McyC are also broadly congruent with the phylogeny of the producer organism (Figure 7a). McyB1 condensation domains were monophyletic and grouped together with condensations domains with a D-peptidyl donor (Figure 7a). Interestingly the McyC condensation domains were also monophyletic but grouped with condensation domains with an L-peptidyl donor (Figure 7a) despite having D-MeAsp as the donor amino acid.

Maximum-likelihood trees based on the amino acid sequence of the condensation domains and the A3–A8 substrate conferring portion of the adenylation domain of McyB1 and McyC differed considerably (Figure 7b). The *mcyB1 *and *mcyC *adenylation domain nucleotide sequences of *Anabaena *spp. 90, 18B6, 66A and *Hapalosiphon hibernicus *BZ-3-1 were all more similar to one another than they were to other *mcyB1 *or *mcyC *sequences (Figure 7b). The nucleotide sequence similarity between each of these pairs of condensation domains was very low and ranged from 27 to 28% (Table 1). However, the nucleotide sequence similarity between each of these pairs of adenylation domains was very high and ranged from 93 to 97% (Table 1). There was no clear evidence for such recent recombination between *mcyB1 *and *mcyC *in *Microcystis viridis *NIES 102, *Planktothrix agardhii *NIVA 126/8, 213 or *Nostoc *sp. 152 (Figure 7b). The sequence divergence between *mcyB1 *and *mcyC *from these strains is higher than the sequence divergence between housekeeping genes and other microcystin synthetase genes within these genera [5,23,24]. In the case of *Nostoc *sp. IO-102-I and *Microcystis aeruginosa *PCC7806 the amino acid sequence of the McyB1 adenylation domain differed considerably from other McyB1 adenylation domains. This region of dissimilarity extended across the entire adenylation domain (A1–A10) in *Nostoc *sp. IO-102-I but was limited to the A3–A8 region of the adenylation domain in *Microcystis aeruginosa *PCC7806. This is reflected in the phylogenetic position of these two adenylation domains in maximum-likelihood trees based on the A3–A8 portions of the adenylation domain (Figure 7b).

**Table 1:** Adenylation and condensation domain divergences

Organism	Strain	Condensation domains	Adenylation domains
*Anabaena *sp.	90	28	95
*Anabaena *sp.	18B6	28	97
*Anabaena *sp.	66A	28	93
*Nostoc *sp.	152	28	80
*Nostoc *sp.	IO-102-I	26	60
*Hapalosiphon hibernicus*	BZ-3-1	27	97
*Planktothrix agardhii*	NIVA126/8	28	70
*Planktothrix agardhii*	213	27	70
*Microcystis aeruginosa*	PCC 7806	27	64
*Microcystis viridis*	NIES 102	28	87

Sequence similarities between nucleotide sequence of the condensation domains from *mcyB1 *and *mcyC *and adenylation domains from *mcyB1 *and *mcyC *from the same strain based on uncorrected p distances converted to percentage similarity.

### Substrate specificities of the mcyB1 and mcyC adenylation domains

The L-Asp residue at position 235 and the L-Lys residue at 517 which interact with the α-amino and the carboxyl groups, respectively, to lock orientation of the L-α-amino acid upon activation [20] were conserved in all strains included in this study (Table 2). The McyB1 and McyC adenylation domain binding pockets differed between 1 and 6 amino acids in pairwise comparisons in most strains (Table 2). However, the amino acids lining the putative binding pockets of McyB1 and McyC in *Anabaena *sp. 18B6 and *Hapalosiphon hibernicus *BZ-3-1 were identical (Table 1).

**Table 2:** Adenylation domain specificity codes

Organism	Strain	Adenyl -ation domain	Binding pocket										LC-MS
				
			235	236	239	278	299	301	322	330	331	517	
*Anabaena *sp.	90	McyB1	D	V	W	F	F	G	L	V	D	K	L-Leu
*Anabaena *sp.	18B6	McyB1	-	-	-	S	-	-	-	-	-	-	L-Arg
*Anabaena *sp.	66A	McyB1	-	-	-	S	-	-	-	-	Y	-	L-Hty
*Nostoc *sp.	152	McyB1	-	A	L	-	-	-	-	I	Y	-	L-Leu
*Nostoc *sp.	IO-102-I	McyB1	-	I	K	N	-	-	A	I	V	-	L-Leu
*Hapalosiphon hibernicus*	BZ-3-1	McyB1	-	-	-	-	-	-	-	-	-	-	L-Leu
*Planktothrix agardhii*	NIVA126/8	McyB1	-	A	L	-	-	-	-	-	-	-	L-Arg
*Planktothrix agardhii*	213	McyB1	-	A	L	-	-	-	-	-	-	-	L-Arg
*Microcystis aeruginosa*	PCC 7806	McyB1	-	A	-	-	L	-	N	N	V	-	L-Leu
*Microcystis viridis*	NIES102	McyB1	-	-	-	T	I	-	A	A	E	-	L-Leu/Arg
													
*Anabaena *sp.	90	McyC	-	-	-	C	-	-	-	-	-	-	L-Arg
*Anabaena *sp.	18B6	McyC	-	-	-	S	-	-	-	-	-	-	L-Arg
*Anabaena *sp.	66A	McyC	-	-	-	S	-	-	-	-	-	-	L-Arg
*Nostoc *sp.	152	McyC	-	-	-	N	-	-	F	I	-	-	L-Arg/Har
*Nostoc *sp.	IO-102-I	McyC	-	-	-	N	-	-	F	-	-	-	L-Arg
*Hapalosiphon hibernicus*	BZ-3-1	McyC	-	-	-	-	-	-	-	-	-	-	L-Ala
*Planktothrix agardhii*	NIVA126/8	McyC	-	P	-	G	-	-	-	-	-	-	L-Arg
*Planktothrix agardhii*	213	McyC	-	P	-	C	-	-	-	-	-	-	L-Arg
*Microcystis aeruginosa*	PCC 7806	McyC	-	-	-	T	I	-	A	-	-	-	L-Arg
*Microcystis viridis*	NIES102	McyC	-	-	-	I	I	-	A	-	-	-	L-Arg

Specificity codes inferred from the protein sequence of the McyB1 and McyC adenylation domains through pairwise alignment against GrsA [20]. The principal amino acid present at the X and Z positions in microcystins analyzed with the LC-MS method.

## Discussion

We did not find separate congruent clusters of McyB1 and McyC adenylation domain sequences (Figures 6, 7) as might have been anticipated under an evolutionary scenario in which all microcystin synthetase genes share the same evolutionary history [14]. Instead we found intermixed clusters of McyB1 and McyC adenylation domains (Figure 7b). In some instances we identified very low levels of sequence divergence in pairwise comparisons of the nucleotide sequences of *mcyB1 *and *mcyC *adenylation domain from the same strain (Table 1). This discordance together with the low levels of sequence divergence is consistent with multiple recent independent recombination events. Recombination would lead to the overwriting of the *mcyB1 *and *mcyC *adenylation domains contributing to sequence homogenization and explain the low divergence of the *mcyB1 *and *mcyC *adenylation domains relative to the *mcyB1 *and *mcyC *condensation domains in *Anabaena *spp. 90, 18B6, 66A and *Hapalosiphon hibernicus *BZ-3-1 (Table 1). However, in addition to these recent recombination events our phylogenetic analysis reveals evidence for replacement of the *mcyB1 *adenylation domain in *Nostoc *sp. IO-102-I and *Microcystis aeruginosa *PCC7806 (Figure 7). The high sequence divergence between the adenylation domains of *mcyB1 *in these two strains and other *mcyB1 *adenylation domain sequences included in this study (Table 1) could be explained by two independent replacement events involving a non-homologous adenylation domain from another peptide synthetase gene cluster. A recombination event has been proposed to replace the adenylation domain of *mcyB1 *and *mcyC *of *Planktothrix agardhii *NIVA 126/8 [12]. However, the McyB1 and McyC adenylation domains of *Planktothrix agardhii *NIVA 126/8 and 213 as well as *Nostoc *sp. 152 clustered separately suggesting that the recombination event precedes the divergence of these two genera. Together, our results indicate that these two adenylation domains are recombination hotspots within the microcystin peptide synthetase gene cluster.

The recombination events at the *mcyB1 *and *mcyC *are limited to the adenylation domain and the condensation domains in *mcyB1 *and *mcyC *are highly divergent and group in separate clusters (Figure 7a). Recombination breakpoints are all limited to the adenylation domain (Figure 4, 5). The phylogenetic discordance between the adenylation and condensation domains is inconsistent with the hypothesis that adenylation and condensation domains are transferred together as a unit. Two rounds of peptide chain elongation are catalyzed by McyB, which typically activates and condenses L-Leu and D-MeAsp into the growing peptide chain [11]. This protein directs the transfer of D-peptidyl intermediates involving a carboxy-terminal epimerase domain of McyA and the condensation domain of McyB1 [11]. Peptide bond formation is achieved between the α-amino group of D-Ala and the α-carboxyl group of L-Leu [11]. In keeping with this the McyB1 condensation domain clusters with domains involved in D-L peptide bonds (Figure 7a). The final condensation reaction is performed between the β-carboxyl group of β-MeAsp and the α-amino group of L-Arg by McyC prior to cyclisation and the resulting peptide bond is atypical [11]. Interestingly, the condensation domain of McyC does not group with previously described D-L condensation domains but group instead with condensation domains with L-peptidyl amino acids as donors (Figure 7a). However, the McyC condensation domain also lacks the typical HHxxxDG his motif in its active site typically present in the condensation domains with D- and L-peptidyl donors [25]. Although adenylation domains are the primary determinants of substrate specificity in NRPSs condensation domains are also reported to exhibit moderate to high substrate selectivity [18]. It may be that differences in the substrate specificities of the condensation domains from McyB1 and McyC mean that the co-transfer of the adenylation and condensation domains would result in a non-functional peptide synthetase. Non-compatible adenylation and condensation domains are predicted to cause a drastic reduction of catalytic competence or even a complete failure to synthesize the desired peptide by the engineered NRPS [21]. Replacement of condensation domains in *mcyB1 *and *mcyC *may lead to a disruption of the overall integrity of the peptide assembly process, in particular the order and timing of condensation reactions.

The simultaneous production of the microcystin -LR and -RR variants has been interpreted as a lack of specificity at the McyB1 adenylation domain [12,13,26]. We predicted the 10 amino acids lining the putative binding pocket in the adenylation domain of McyB1 and McyC though alignment against the GrsA adenylation domain [20]. The 8 amino acids lining the binding pocket which interact with the side chain and functional group and were highly variable in McyB1 and to a lesser extent McyC (Table 2). Single amino acid changes in the amino acids lining the putative binding pocket of the adenylation domain are known to have an effect on the type of amino acid that is recognized and activated by the adenylation domain [20]. Single amino acid change (V→I) in McyC could be responsible for shifting the incorporation of L-Arg towards L-Har in *Nostoc *sp. 152 (Table 2). Similarly a single amino acid change in (D→Y) in McyB1 could be responsible for shifting the incorporation of L-Arg towards L-Hty in *Anabaena *sp. 66A. Experimental expression and mutation of the adenylation domains in each case could verify this hypothesis.

Determining the amino acids lining the substrate conferring portions of the adenylation domains of non-ribosomal peptide synthetase gene clusters can yield invaluable predictions of substrate specificities of unknown peptide synthetases [27]. The putative binding pocket of the adenylation domains of McyB1 and McyC in *Hapalosiphon hibernicus *BZ-3-1 are identical (Table 2). However, this strain incorporated 91% L-Leu at the X position and 99% L-Ala at the Z position (Figure 3). Our results suggest that caution should be taken when inferring substrate specificity given the general lack of knowledge about how widespread adenylation domain replacement is in nature.

Many important antibiotics, antimicrobial compounds, siderophores and toxins are synthesized on non-ribosomal peptide synthetase enzyme complexes [16]. There is much current interest in engineering non-ribosomal peptide synthetases in order to create new peptides with potential biological activities [17]. It has been suggested that peptide synthetase would gain most effectively through transfer of entire modules [18,21]. Some artificial combinations of adenylation and condensation domains result in non-functional products [21]. This led to the hypothesis that non-ribosomal peptide synthetase modules evolve as a unit [18]. Here we have clear evidence for the exchange and replacement of the adenylation domain without the concomitant transfer of the condensation domain.

## Conclusion

Our results demonstrate that the *mcyB1 *and *mcyC *adenylation domains are recombination hotspots in the microcystin synthetase gene cluster. We show clear evidence for the recurrent exchange and replacement of the adenylation domain in a broad range of microcystin producing cyanobacteria. Our results show that functional peptide synthetases can be created in nature through transfer of adenylation domains without the concomitant transfer of condensation domains.

## Methods

### Taxon sampling and LC-MS

We selected representative producers of microcystins from the genera *Anabaena, Hapalosiphon, Microcystis*, *Nostoc*, and *Planktothrix *(see additional file 1). To obtain sufficient biomass for LC-MS analysis 10 cyanobacterial strains were grown at a photon irradiance of 20–27 μmol m^-2 ^sec^-1 ^in 2.7 liters of Z8 medium aerated with filter sterilized compressed air. Cells from 21 day old cultures were homogenized with 425–1180 μm diameter glass beads and 1 ml of 85% acetonitrile. The mixture was shaken in a FP120 FastPrep cell disruptor (Savant Instruments Inc.) and then centrifuged at 10,000 × g for 3 min. The supernatant was passed sequentially through two-solid phase extraction cartridges (StrataX Polymeric Sorbent) equilibrated with 1 ml of 85% acetonitrile and a 0.2 μm pore-size filter (GHP Acrodisc).

Microcystins were analyzed by injecting 10 μl of this extract into an Agilent 1100 series modular HPLC system (Agilent technologies) equipped with a diode array detector and a mass spectrometer (Agilent XCT Plus Ion Trap). A Luna-C18 (2) column (5 μm, 2 × 150 mm, Phenomenex) at 40°C and a mobile phase gradient of 5% (0 min) to 100% (50 min) isopropanol (+0.1% formic acid) in 0.1% formic acid at a flow rate of 0.15 ml min^-1 ^were used. Microcystins were distinguished from other peptides based on their characteristic UV maximum absorbance at 238 nm and on their mass spectral characteristics as MH^+ ^values corresponding to the range of published microcystins, loss of neutral fragment 134 in the ion source, occurrence of ions *m/z *585/599/627/641 [(MeAsp)-(H)ar-(DM/ADM)Adda-(Glu)+H^+^] and *m/z *375/361 (Adda -134-Glu-(M)dha) in the MS^2 ^spectrum. Comparison of LC-MS properties of reference strain microcystins aided assignment of structure to the microcystin. Electrospray ionization was performed in positive ion mode. Nebulizer gas (N_2_) pressure was 30–50 psi (lb/in^2^) (207–345 kPa), drying gas flow and temperature 8–12 L min^-1 ^and 350°C, respectively. The capillary voltage was set to 3270 V, capillary exit offset to 317 V, skimmer 1 potential to 41.5 V with a trap drive value 82.8. Spectra were recorded as averages of 4 using ultra scan mode and a scan range from 50 to 1200 *m/z*. MS^2 ^spectra were recorded as averages of 3 with manual and auto MS mode with fragmentation amplitude of 0.50 V. In auto MS mode 5 precursor ions from ion range 800 – 1200 m/z were detected with an isolation width of 4.0 m/z.

### PCR and sequencing

Total genomic DNA was extracted from 40 ml of cyanobacterial cultures using a hot phenol method [28]. We amplified portions of the 16S rRNA, *rpoC1*, *rpoB, tufA*, and *rbcL *genes using sets of specific oligonucleotide primers (see additional file 1). These 5 housekeeping genes are present in all cyanobacteria and are thought to be largely unaffected by horizontal gene transfer. PCR reactions were performed in a 20 μl final volume containing approximately 20–100 ng of DNA, 1 × DynaZyme II PCR buffer, 250 μM of each deoxynucleotide, 0.5 μM of each oligonucleotide primer, and 0.5 units of DynaZyme II DNA polymerase (Finnzymes, Espoo, Finland). The following protocol was used: 95°C for 3 min; 30 cycles of denaturation at 94°C for 30 sec, annealing at 56°C for 30 sec and elongation at 72°C for 1 min, followed by a final elongation of 72°C for 10 min. To study the evolution of the microcystin biosynthetic system in these strains we chose 5 regions of the microcystin synthetase gene cluster, *mcyD, mcyE, mcyG, mcyB *and *mcyC *using sets of specific oligonucleotide primers (see additional file 1). PCR reactions were performed as before but with primer concentration increased to 0.7 μM and a 3-minute elongation time to amplify the 3.5 kb *mcyB *and *mcyC *PCR products. The size of the PCR amplification products was checked in agarose gels and PCR products were purified using Montage™ PCR Centrifugal Filter Devices (Millipore, Billerica, MA, USA). The purified PCR products were Sanger sequenced with the external primers used in PCR and where necessary sets of internal primers (see additional file 1). Cycle sequencing products were purified and separated on an ABI PRISM 310 Genetic Analyzer. Chromatograms were checked and edited with CHROMAS 2.2 program (Technelysium Pty Ltd.). Contig assembly and alignment of the sequences were performed with the BIOEDIT Sequence Alignment Editor.

### Detection of recombination

We screened *mcyB1 *and *mcyC *sequences using the program TREEORDERSCAN [29]. The TREEORDERSCAN program provides a rapid method to detect intergenotype recombination among individual sequences. Based on the alignment of *mcyB1 *and *mcyC *genes from 10 strains of toxic cyanobacteria, the phylogenetic compatibility matrix was constructed through comparing congruence between subtrees of whole alignment. At first, 67 alignment fragments were obtained by moving a 300 nucleotide window along the alignment with a step of 50 bases, and neighbor-joining tree of each fragment was constructed by PHYLIP [30]. Then phylogenetic violations of any two different subtrees were calculated by TREEORDERSCAN (Simmonic 2005 version 1.5), and presented proportionally as a colour gradient.

Detection of potential recombinant sequences, identification of likely parent sequences, and localization of possible recombination breakpoints were done in RDP3 [31]. The RDP3 package uses a mixture of statistical and phylogenetic methods to both identify probable recombination events within individual sequences and a minimal subset of unique events detectable within an entire alignment. To investigate the extent of recombination within the data set, the aligned sequences were examined using RDP3 [31], GENECONV [32], BOOTSCAN [33], MAXIMUM CHI SQUARE [34], CHIMAERA [33], and SISTER SCAN [35] recombination detection methods as implemented in RDP3 [33]. Standard settings in RDP3 for all methods were that sequences were considered as linear, the P-value cutoff was set to 0.05, the standard Bonferroni correction was used, consensus daughters were found and breakpoints were polished. With the set of unique recombination events identified by these 6 detection methods a breakpoint map containing the positions of all positively identified breakpoints was constructed by moving a 200 nucelotide window and counting all the identified breakpoints falling within each window. A breakpoint density graph was created by plotting these numbers at the position of the centre of the window. For each window, a permutation test was made for breakpoint clustering analysis and to define the thresholds areas.

### Phylogenetic analyses

We investigated competing hypotheses concerning the origin and timing of the recombination events in the microcystin synthetase gene cluster by reconstructing the evolutionary history of both the microcystin synthetase gene cluster and the producer organisms. We amplified and sequenced portions of genes from both the microcystin synthetase gene cluster and housekeeping genes. In order to reconstruct the evolutionary history of the microcystin synthetase gene cluster we assembled a 3199 bp data set comprised of a mixed polyketide synthase/peptide synthetase gene (*mcyE*) and polyketide synthase genes (*mcyD *and *mcyG*) (see additional file 1). The phylogenetic analysis was rooted as described previously using homologues identified in BLAST (blastp) searches [14]. We used random taxon addition (10 replicates), tree-bisection-reconnection branch-swapping, and heuristic searches with 100,000 repartitions of the data. The data from all 3 genes was concatenated in order to increase the amount of information available in phylogenetic analyses. We reconstructed the evolutionary history of the producer organisms by assembling a 3586 bp data set comprised of 16S rRNA, *rpoB*, *rpoC1, tufA *and *rbcL *gene sequences (see additional file 1). These genes are involved in carbon fixation, transcription and translation, conserved and widely used as tools for phylogenetic classification. The 16S rRNA, *rpoB, tufA, rpoC1 *and *rbcL *gene sequences of the early branching cyanobacteria *Gloeobacter violaceus *PCC 7421 (BA000045) and *Thermosynechococcus elongatus *BP-1 (BA000039) were used as outgroups. The 16S rRNA, *rpoB, tufA, rpoC1 *and *rbcL *sequence data were concatenated into a single data set. Phylogenetic analyses of these two datasets were conducted using PAUP*4.0 [36]. Priming sites and ambiguous regions of the alignment were excluded. Phylogenetic trees were inferred using maximum-likelihood optimization criteria. Maximum-likelihood analyses were performed with ten heuristic searches, random addition-sequence starting trees, and tree bisection and reconnection branch arrangements. The GTR model of DNA substitution with a gamma distribution of rates and constant sites removed in proportion to base frequencies was used in maximum-likelihood analyses. We analyzed 1000 bootstrap replicates to test the stability of monophyletic groups.

In order to investigate recombination between the adenylation domain of *mcyB1 *and *mcyC *we obtained sequence data from *mcyB1 *(3494–3566 bp) and *mcyC *(3581–3593 bp). The *mcyB1 *PCR product contained the condensation, adenylation and thiolation domains of the first module as well as a fragment of the condensation domain from the second module. The *mcyC *gene PCR product contained the condensation, adenylation, thiolation domain as well as part of the thioesterase domain. Sequence data was partitioned into adenylation and condensation domain sequences and analyzed separately. We obtained a selection of condensation and adenylation domains from NCBI and aligned them against the McyB1 and McyC adenylation and condensation domains amino acid sequences in BIOEDIT (see additional file 1). Regions of ambiguous alignment were excluded and we considered 352 aa of the condensation domain and 197 aa of the adenylation domain (A3–A8) for phylogenetic analyses. Protein maximum-likelihood phylogenies of each dataset were inferred using PROML implemented in the PHYLIP 3.6 package [30] with a JTT substitution model. Ten random additions with global rearrangements were used to find the optimal tree. We performed 1,000 distance bootstrap replicates using the SEQBOOT, PROTDIST (JTT substitution model), and CONSENSE programs of the PHYLIP 3.6 package [30].

### Substrate specificities of the mcyB1 and mcyC adenylation domains

Manual alignment against the GrsA primary amino acid sequence between the core motifs A4 and A10 allowed extraction of the 10 amino acids predicted to line the binding pocket of the adenylation domain for both McyB1 and McyC [20]. According to this model, the L-Asp residue at position 235 and the L-Lys residue at 517 interact with the α-amino and the carboxyl groups, respectively, to lock orientation of the L-α-amino acid upon activation [20]. This configuration projects the side chain of the amino acid into the binding pocket where it is bound by the remaining 8 amino acids lining the pocket. Manual substrate specificities predictions were confirmed using the automated NRPSpredictor tool [37].

## Abbreviations

D-MeAsp, D-*erythro*-β-methyl-aspartic acid; 

Adda, (2S,3S,8S,9S) 3-amino-9-methoxy-2,6,8-trimethyl-10-phenyl-(4*E*), (6*E*)-decadienoic acid; 

D-Glu, D-*iso*-glutamic acid; 

Mdha, *N*-methyldehydroalanine; 

Hty, homotyrosine; 

Hil, homoisoleucine; 

Aba, Aminoisobutyric acid; 

Har, homoarginine; 

Hph, homophenylalanine.

## Competing interests

The author(s) declares that there are no competing interests.

## Authors' contributions

DPF conceived of the study, carried out the molecular genetic studies, participated in the sequence alignment, performed the phylogenetic analysis and drafted the manuscript. LR conceived of the study and drafted the manuscript. JJ performed the LC-MS analysis and drafted the manuscript. MW performed the LC-MS analysis. HW performed the recombination analysis. KL carried out the molecular genetic studies and drafted the manuscript. KS participated in its design and coordination and helped to draft the manuscript. All authors read and approved the final manuscript.

## Supplementary Material

Additional file 1 Further supplementary information on materials and methods used in this study as well as data on the different types of microcystin variants produced by the cyanobacterial strains. Click here for file
