# Supplementary material for: Recurrent adenylation domain replacement in the microcystin synthetase gene cluster
Source: BMC Evol Biol. 2007 Oct 1;7:183. doi: 10.1186/1471-2148-7-183 (PMC2174460; doi:10.1186/1471-2148-7-183)
Supplement: Additional file 1 — Further supplementary information on materials and methods used in this study as well as data on the different types of microcystin variants produced by the cyanobacterial strains. [file 1471-2148-7-183-S1.doc]

Additional file 1

Recurrent adenylation domain replacement in the microcystin synthetase gene cluster

David P. Fewer, Leo Rouhiainen, Jouni Jokela, Matti Wahlsten, Kati Laakso, Hao Wang, and Kaarina Sivonen*

Department of Applied Chemistry and Microbiology, P.O. Box 56, Viikki Biocenter, Viikinkaari 9, FIN-00014, University of Helsinki, Finland

^*^ Corresponding author:

Kaarina Sivonen

Department of Applied Chemistry and Microbiology, P.O. Box 56, Viikki Biocenter, Viikinkaari 9, FIN-00014, University of Helsinki, Finland

Tel. +358-9-19159270

Fax. +358-9-19159322

Email: kaarina.sivonen@helsinki.fi

**Supplementary Tables (S1-S3)**

**Table S1 - Strains used in this study**

The cyanobacterial strains used in this study including strain number, year and place of isolation and accession numbers for the microcystin synthetase genes and housekeeping genes used in the phylogenetic analysis. Accession numbers in bold denote sequences that were obtained for this study. NIVA, Culture Collection of Algae, Norway; PCC, Pasteur Culture Collection, France; NIES, National Institute for Environmental Studies Microbial Culture Collection, Japan.

| Organism | Strain | Year | Origin | Microcystin syntethase genes | | | | |  | Housekeeping genes | | | | |
| --- | --- | --- | --- | --- | --- | --- | --- | --- | --- | --- | --- | --- | --- | --- |
|  |  |  |  | *mcyB* | *mcyC* | *mcyD* | *mcyE* | *mcyG* |  | 16S | *rpoB* | *rpoC1* | *tufA* | *rbcL* |
|  |  |  |  |  |  |  |  |  |  |  |  |  |  |  |
| *Hapalosiphon hibernicus* | BZ-3-1 | 1984 | Hawaii | EU151872 | EU151865 | EU151879 | EU151886 | EU151893 |  | EU151900 | EU151903 | EU151909 | EU151914 | EU151924 |
| *Anabaena* sp. | 90 | 1986 | Finland | AJ536156 | AJ536156 | AJ536156 | AJ536156 | AJ536156 |  | AJ133156 | AJ628124 | AY424996 | EU151915 | EU151925 |
| *Anabaena* sp. | 18B6 | 1986 | Finland | EU151873 | EU151866 | EU151880 | EU151887 | EU151894 |  | EU151901 | EU151904 | EU151910 | EU151916 | EU151926 |
| *Anabaena* sp. | 66A | 1986 | Finland | EU151874 | EU151867 | EU151881 | EU151888 | EU151895 |  | AJ133157 | AJ628126 | EU151911 | EU151917 | EU151927 |
| *Nostoc* sp. | 152 | 1986 | Finland | EU151875 | EU151868 | EU151882 | EU151889 | EU151896 |  | AJ133161 | AJ628129 | AY424997 | EU151918 | EU151928 |
| *Nostoc* sp. | IO-102-I | 2000 | Finland | EU151876 | EU151869 | EU151883 | EU151890 | EU151897 |  | AY566855 | EU151905 | AY566859 | EU151919 | EU151929 |
| *Planktothrix agardhii* | 213 | 1987 | Finland | EU151877 | EU151870 | EU151884 | EU151891 | EU151898 |  | EU151902 | EU151906 | EU151912 | EU151920 | EU151930 |
| *Planktothrix agardhii* | NIVA126/8 | 1984 | Finland | AJ441056 | AJ441056 | AJ441056 | AJ441056 | AJ441056 |  | AJ133166 | AJ628133 | EU151913 | EU151921 | EU151931 |
| *Microcystis aeruginosa* | PCC 7806 | 1972 | Holland | AF183408 | AF183408 | AF183408 | AF183408 | AF183408 |  | AF139299 | EU151907 | AY425000 | EU151922 | AM157793 |
| *Microcystis viridis* | NIES 102 | 1982 | Japan | EU151878 | EU151871 | EU151885 | EU151892 | EU151899 |  | D89033 | EU151908 | AY425001 | EU151923 | EU151932 |
|  |  |  |  |  |  |  |  |  |  |  |  |  |  |  |

NOTE *Hapalosiphon hibernicus* BZ-3-1 was obtained from the Patterson Culture Collection of the MarBEC culture collections. The remaining strains are maintained in our own culture collection at the University of Helsinki.

**Table S2. Sets of oligonucletoide primers used for PCR amplification and sequencing of gene fragments**

PCR primers used in this study, listing the target, sequence, melting temperature and origin of the primers. External primers were used in PCR and sanger sequencing and internal primers used to finish the sequence of the larger PCR products.

| Primer | Target | Sequence (5'- 3') | Tm (°C) | Use | Reference |
| --- | --- | --- | --- | --- | --- |
|  |  |  |  |  |  |
| pA | 16S rRNA | gagtttgatcctggctcag | 60.5 | External | Edwards et al. 1989 |
| B23S | 23S rRNA | cttcgcctctgtgtgcctaggt | 68.9 | External | Lepère et al., 2000 |
| 16S545R | 16S rRNA | attccggataacgcttgc | 63.0 | Internal | Rajaniemi et al. 2005 |
| 16S1092R | 16S rRNA | gcgctcgttgcgggactt | 71.3 | Internal | Rajaniemi et al. 2005 |
| 16S979F | 16S rRNA | cgatgcaacgcgaagaac | 66.0 | Internal | Rajaniemi et al. 2005 |
| RF | *rpoC1* | tgggghgaaagnacaytncctaa | 53.2 | External | Rantala et al. 2004 |
| RR | *rpoC1* | gcaaancgtccnccatcyaaytgba | 58.5 | External | Rantala et al. 2004 |
| rpoBF | *rpoB* | gtagttgtarccntccca | 44.2 | External | Rajaniemi et al. 2005 |
| rpoBR | *rpoB* | rcmgcmgacgaagaagacg | 50.0 | External | Rajaniemi et al. 2005 |
| rbcLF | *rbcL* | gacttcaccaaagaygacgaaaacat | 65.0 | External | This study |
| rbcLR | *rbcL* | gaactcgaacttratytctttcca | 57.9 | External | This study |
| TF | *tufA* | cacgtdgaytgyccnggncacgctg | 63.8 | External | This study |
| TR | *tufA* | atncgrtcnccdggcataaccatttc | 60.2 | External | This study |
| DF | *mcyD* | gctcaagaaaaattattacatcaag | 59.2 | External | This study |
| DR | *mcyD* | ttaaaggagaatgaaaagcatgaga | 64.0 | External | This study |
| mcyDF | *mcyD* | gatccgattgaattagaaag | 55.9 | Internal | Rantala et al. 2004 |
| mcyDR | *mcyD* | gtattccccaagattgcc | 59.8 | Internal | Rantala et al. 2004 |
| EF | *mcyE* | catcdraratratrtggtgcatat | 44.7 | External | This study |
| ER | *mcyE* | ggiwcdacigghaaicctaaagg | 50.9 | External | This study |
| mcyEF2 | *mcyE* | gaaatttgtgtagaaggtgc | 56.8 | Internal | Rantala et al. 2004 |
| mcyER4 | *mcyE* | aattctaaagcccaaagacg | 60.2 | Internal | Rantala et al. 2004 |
| mcyGF | *mcyG* | gaaattggtgcgggaactggag | 70.7 | External | This study |
| mcyGR | *mcyG* | tttgagcaacaatgatactttgctg | 66.1 | External | This study |
| pB1F | *mcyB* | GCAGGAAGGGATGCTCTTTCATAG | 68.0 | Internal | This study |
| pB2F | *mcyB* | CATCATATTHTDTTGGATGGTTGG | 60.5 | External | This study |
| pB3F | *mcyB* | CAAAARCARGCAGAAMTTCAGG | 56.1 | Internal | This study |
| pB4F | *mcyB* | GTGTTTAGATAGBGATTGG | 44.4 | Internal | This study |
| pB5F | *mcyB* | GGMGAATTACATATTGG | 42.5 | Internal | This study |
| pB6R | *mcyB* | CCATCACAAATAATATGATG | 53.2 | External | This study |
| pB7R | *mcyB* | TGACTAGAGHAATDGCTTT | 40.6 | Internal | This study |
| pB9R | *mcyB* | CCAATCVCTATCTAAACAC | 44.4 | Internal | This study |
| pB8R | *mcyB* | CCAATATGTAATTCKCC | 42.5 | Internal | This study |
| pB10R | *mcyB* | CCAACCATCCAAHADAATATGATG | 60.5 | Internal | This study |
| pC1F | *mcyC* | GGMTKTGGATTYTGCASCATAT | 49.0 | External | This study |
| pC2F | *mcyC* | GCAAAATCAGTGGTTAGAAAGTGA | 63.4 | Internal | This study |
| pC3F | *mcyC* | TATAGTGATTATCCTTTTGATAA | 52.3 | Internal | This study |
| pC4F | *mcyC* | TGTGTTTAGATAGGGATTGG | 57.0 | Internal | This study |
| pC5F | *mcyC* | AAACCTGAAGGTCATAAAGATA | 56.5 | Internal | This study |
| pC6F | *mcyC* | ATTGAATTAGGAGAAAT | 45.7 | Internal | This study |
| pC7F | *mcyC* | CCTTTATCAATGATTTATGAAAA | 56.9 | Internal | This study |
| pC8R | *mcyC* | CCAGAGAAAATTAAAGTATTCCCA | 61.4 | External | This study |
| pC9R | *mcyC* | ATTGATAAAGGAATTTC | 46.2 | Internal | This study |
| pC10R | *mcyC* | CCAATATGTAATTCTCC | 47.4 | Internal | This study |
| pC11R | *mcyC* | TTTATGACCTTCAGGTTTAGC | 57.9 | Internal | This study |
| pC12R | *mcyC* | TATATTCATAAGCATCAA | 46.4 | Internal | This study |
| pC13R | *mcyC* | CACTTTCTAACCACTGATTTTGCCA | 67.0 | Internal | This study |
|  |  |  |  |  |  |

NOTE - In cases where sequencing primers failed to generate reliable sequence we designed specific primers to finish the sequence.

**References**

**Rantala A, Fewer DP, Hisbergues M, Rouhiainen L, Vaitomaa J, Börner T, Sivonen K:** **Phylogenetic evidence for the early evolution of microcystin synthesis.** *Proc. Natl. Acad. Sci. USA.* 2004, 101**:** 568-573

Lepère C, Wilmotte A, Meyer B: **Molecular diversity of *Microcystis* strains (Cyanophyceae, Chroococcales) based on 16S rDNA sequences**. Syst. Geogr. 2000, 70: 275–283

Rajaniemi P, Hrouzek P, Kastovska K, Willame R, Rantala A, Hoffmann L, Komarek J, Sivonen K**: Phylogenetic and morphological evaluation of the genera *Anabaena*, *Aphanizomenon*, *Trichormus* and *Nostoc* (Nostocales, Cyanobacteria).** *Int. J. Syst. Evol. Microbiol*. 2005, 55: 11-26.

**Table S3. Organism, strain, gene cluster, product and DDBJ/EMBL/GenBank Accession numbers for sequences used to construct the maximum likelihood phylogenies in Figure 7.**

| Organism | Strain | Product | Gene cluster | Accession |
| --- | --- | --- | --- | --- |
|  |  |  |  |  |
| *Anabaena circinalis* | 90 | Anabaenopeptilides | Apd | AJ269505 |
| *Pseudomonas* sp. | MIS38 | Arthrofactin | Arf | AB107223 |
| *Bacillus licheniformis* | A ATCC0716 | Bacitracin | Bac | AF007865 |
| *Streptomyces lavendulae* | ? | Complestatin | Com | AF386507 |
| *Bacillus subtilis* | F29-3 | Fengycin | Fen | AF023464 |
| *Brevibacillus brevis* | ATCC 9999 | Gramicidin | Grs | X61658 |
| *Bacillus subtilis* | RB14 | Iturin A | Itu | AB050629 |
| *Bacillus licheniformis* | ATCC0716 | Lichenysin | Lic | U95370 |
| *Nostoc* sp*.* | GSV224 | Nostopeptolide | Nos | AF204805 |
| *Pseudomonas aeruginosa* | PAO | Pyoverdin D | Pvd | U07359 |
| *Pseudomonas syringae* | DC3000 | Pyoverdin S | Pvs | AE016863 |
| *Bacillus subtilis* | W168 | Surfactin | Srf | X70356 |
| *Pseudomonas syringae* | B310D | Syringopeptin22 | Syp | AF286216 |
| *Brevibacillus brevis* | ATCC 8185 | Tyrocidine | Tyc | AF004835 |
|  |  |  |  |  |

**Table S4. Structures, molecular ion masses, identified amino acids in positions X and Z in LC-MS results, and relative proportions (%) of microcystins within the strains.**

Hil, homoisoleucine; Har, homoarginine; Hph, homophenylalanine; Hty, homotyrosine, and X is an unknown amino acid(s).

| Strain | Microcystin | [M+H]^+^ (m/z) | Variable amino acid in position | | Relative microcystin % within the strain |
| --- | --- | --- | --- | --- | --- |
|  |  |  | X | Z |  |
| *Anabaena* sp. 90 | |  |  |  |  |
| 1 | [D-Asp^3^]mcyst-RR | 1,024 | R | R | 2.3 |
| 2 | Mcyst-RR | 1,038 | R | R | 5.4 |
| 3 | [D-Asp^3^, MeSer^7^]mcyst-LR | 999 | L | R | 0.1 |
| 4 | [DMAdda^5^]mcyst-LR | 981 | L | R | 0.2 |
| 5 | [MeSer^7^]mcyst-LR | 1,013 | L | R | 0.8 |
| 6 | Mcyst-LR | 995 | L | R | 54.5 |
| 7 | [D-Asp^3^]mcyst-LR | 981 | L | R | 34.1 |
| 8 | [Dha^7^]mcyst-LR | 981 | L | R | 0.0 |
| 9 | [D-Asp^3^]mcyst-HilR | 995 | Hil | R | 0.9 |
| 10 | Mcyst-HilR | 1,009 | Hil | R | 1.5 |
| 11 | Unknown microcystin | 997 |  |  | 0.2 |
| *Anabaena* sp. 18B6 | |  |  |  |  |
| 1 | [X]mcyst-RR | 1,028 | R | R | 1.2 |
| 2 | [D-Asp^3^, Dha^7^]mcyst-RR | 1,010 | R | R | 78.8 |
| 3 | demethyl-mcyst-RR | 1,024 | R | R | 19.9 |
| 4 | Mcyst-XR | 1,031 |  | R | 0.1 |
| *Anabaena* sp. 66A | |  |  |  |  |
| 1 | [DMAdda^5^,(M)dha^7^]mcyst-(H)tyR | 1,031 | Hty/Y | R | 0.02 |
| 2 | [D-Asp^3^,Dha^7^]mcyst-XR | 1,031 |  | R | 0.05 |
| 3 | [X]Mcyst-HtyR | 1,045 | Hty | R | 0.03 |
| 4 | [D-Asp^3^]mcyst-XR | 1,001 |  | R | 0.02 |
| 5 | [X]Mcyst-(H)tyR | 1,045 | Hty/Y | R | 0.1 |
| 6 | Mcyst-XR | 1,015 |  | R | 0.05 |
| 7 | [X]Mcyst-X/HtyR | 1,031 | Hty | R | 0.2 |
| 8 | [X]Mcyst-(H)tyR | 1,045 | Hty/Y | R | 0.1 |
| 9 | [D-Asp^3^, L-Ser^7^]mcyst-HtyR | 1,049 | Hty | R | 2.3 |
| 10 | [L-Ser^7^]mcyst-HtyR | 1,063 | Hty | R | 4.2 |
| 11 | [D-Asp^3^,Dha^7^]mcyst-HtyR | 1,031 | Hty | R | 28.6 |
| 12 | [Dha^7^]mcyst-HtyR | 1,045 | Hty | R | 56.5 |
| 13 | Mcyst-HtyR | 1,059 | Hty | R | 0.1 |
| 14 | [X, L-Ser^7^]mcyst-LR | 971 | L | R | 0.1 |
| 15 | [D-Asp^3^]mcyst-XR | 983 |  | R | 0.04 |
| 16 | [Dha^7^]mcyst-LR | 981 | L | R | 1.8 |
| 17 | demethyl-[L-Ser^7^]mcyst-LR | 985 | L | R | 0.1 |
| 18 | [L-Ser^7^]mcyst-LR | 999 | L | R | 0.1 |
| 19 | [Dha^7^ ]mcyst-FR | 1,015 | F | R | 0.6 |
| 20 | [D-Asp^3^,Dha^7^]mcyst-LR | 967 | L | R | 1.2 |
| 21 | [D-Asp^3^,Dha^7^]mcyst-FR | 1,001 | F | R | 0.2 |
| 22 | [X]Mcyst-LR | 995 | L | R | 0.03 |
| 23 | [Dha^7^]mcyst-HphR | 1,029 | Hph | R | 1.7 |
| 24 | [D-Asp^3^,Dha^7^]mcyst-HphR | 1,015 | Hph | R | 0.8 |
| 25 | Unknown microcystin | 1,049 |  |  | 0.03 |
| 26 | Unknown microcystin | 1,063 |  |  | 0.1 |
| 27 | Unknown microcystin | 1,049 |  |  | 0.1 |
| 28 | Unknown microcystin | 1,063 |  |  | 0.1 |
| 29 | Unknown microcystin | 1,047 |  |  | 0.2 |
| 30 | Unknown microcystin | 1,061 |  |  | 0.2 |
| 31 | Unknown microcystin | 1,035 |  |  | 0.2 |
| 32 | Unknown microcystin | 1,047 |  |  | 0.1 |
| 33 | Unknown microcystin | 1,047 |  |  | 0.1 |
| *Nostoc* sp. 152 |  |  |  |  |  |
| 1 | [X]mcyst-LR | 1,039 | L | R | 0.1 |
| 2 | [D-Asp^3^, DMAdda^5^]mcyst-LR | 967 | L | R | 0.0 |
| 3 | [DMAdda^5^]mcyst-LR | 981 | L | R | 0.2 |
| 4 | [DMAdda^5^]mcyst-LHar | 995 | L | Har | 0.1 |
| 5 | [ADMAdda^5^]mcyst-XR | 1,023 |  | R | 0.0 |
| 6 | [X, D-Asp^3^]mcyst-HilR | 1,039 | Hil | R | 0.2 |
| 7 | [D-Asp^3^, ADMAdda^5^]mcyst-VR | 995 | V | R | 0.0 |
| 8 | [D-Asp^3^, ADMAdda^5^, Dha^7^]mcyst-HilR | 1,009 | Hil | R | 0.0 |
| 9 | [X, ADMAdda^5^]mcyst-LR | 1,023 | L | R | 0.1 |
| 10 | [X, ADMAdda^5^]mcyst-LHar | 1,037 | L | Har | 0.1 |
| 11 | [ADMAdda^5^, Dha^7^]mcyst-LR | 1,009 | L | R | 0.3 |
| 12 | [X, D-Asp^3^]mcyst-LR | 995 | L | R | 0.2 |
| 13 | [Ser^1^, D-Asp^3^, ADMAdda^5^]mcyst-LR | 1,039 | L | R | 0.3 |
| 14 | [X, D-Asp^3^, ADMAdda^5^]mcyst-LR | 1,009 | L | R | 0.7 |
| 15 | [X, D-Asp^3^, ADMAdda^5^]mcyst-LR | 1,009 | L | R | 8.7 |
| 16 | [ADMAdda^5^]mcyst-LR | 1,023 | L | R | 44.0 |
| 17 | [ADMAdda^5^]mcyst-LHar | 1,037 | L | Har | 40.3 |
| 18 | [X, D-Asp^3^, ADMAdda^5^]mcyst-LR | 1,009 | L | R | 1.6 |
| 19 | [D-Asp^3^, ADMAdda^5^, Dha^7^]mcyst-LR | 995 | L | R | 0.3 |
| 20 | [ADMAdda^5^]mcyst-HilR | 1,037 | Hil | R | 1.7 |
| 21 | [ADMAdda^5^]mcyst-HilHar | 1,051 | Hil | Har | 1.0 |
| *Nostoc* sp. IO-102-I |  |  |  |  |  |
| 1 | [DMAdda^5^]mcyst-LR | 981 | L | R | 2.0 |
| 2 | [DMAdda^5^]mcyst-HilR | 995 | Hil | R | 0.1 |
| 3 | [ADMAdda^5^]mcyst-XR | 1,095 |  | R | 0.1 |
| 4 | [ADMAdda^5^]mcyst-XR | 1,077 |  | R | 4.3 |
| 5 | [ADMAdda^5^]mcyst-XR | 1,063 |  | R | 0.1 |
| 6 | [ADMAdda^5^]mcyst-YR | 1,073 | Y | R | 0.3 |
| 7 | [ADMAdda^5^]mcyst-XR | 1,075 |  | R | 0.2 |
| 8 | [X]mcyst-LR | 1,009 | L | R | 0.2 |
| 9 | Mcyst-XR | 1,041 |  | R | 0.1 |
| 10 | [X]mcyst-LR | 1,009 | L | R | 0.2 |
| 11 | [D-Asp^3^, ADMAdda^5^]MCYST-LR | 1,009 | L | R | 2.6 |
| 12 | [ADMAdda^5^]mcyst-LR | 1,023 | L | R | 82.2 |
| 13 | Mcyst-XR | 1,009 |  | R | 1.2 |
| 14 | Mcyst-XR | 1,041 |  | R | 1.2 |
| 15 | Mcyst-XR | 995 |  | R | 0.1 |
| 16 | [(X), ADMAdda^5^]mcyst-(F)R | 1,057 | (F) | R | 1.1 |
| 17 | Mcyst-XR | 1,038 |  | R | 1.2 |
| 18 | [(X), DMAdda^5^]mcyst-(Hil)R | 1,037 | (Hil) | R | 1.3 |
| 19 | Unknown microcystin | 1,037 |  |  | 1.7 |
| 20 | Unknown microcystin | 1,037 |  |  | 0.0 |
| *Hapalosiphon hibernicus* BZ-3-1 | |  |  |  |  |
| 1 | [D-Asp3, DMAdda5]mcyst-LA | 882 | L | A | 1.3 |
| 2 | [D-Asp3]mcyst-RA | 939 | R | A | 1.6 |
| 3 | Mcyst-RA | 953 | R | A | 6.7 |
| 4 | [D-Asp3]mcyst-VA | 882 | V | A | 0.5 |
| 5 | Mcyst-VA | 896 | L | A | 1.5 |
| 6 | [D-Asp3]mcyst-LA | 896 | L | A | 15.9 |
| 7 | Mcyst-LA | 910 | L | A | 70.1 |
| 8 | [Dha7]mcyst-LA | 896 | L | A | 1.0 |
| 9 | [D-Asp3]mcyst-LV | 924 | L | V | 0.4 |
| 10 | Mcyst-LV | 938 | L | V | 0.4 |
| 11 | Mcyst-LL | 952 | L | L | 0.6 |
| *Planktothrix agardhii* NIVA 126/8 | |  |  |  |  |
| 1 | [D-Asp^3^]mcyst-RR | 1,024 | R | R | 68.9 |
| 2 | [D-Asp^3^]mcyst-LR | 981 | L | R | 30.5 |
| 3 | Unknown microcystin | 967 |  |  | 0.6 |
| *Planktothrix agardhii* NIVA 126/8 | |  |  |  |  |
| 1 | [D-Asp^3^]mcyst-RR | 1,024 | R | R | 83.8 |
| 2 | [D-Asp^3^]mcyst-LR | 981 | L | R | 15.4 |
| 3 | [D-Asp^3^, Dha^7^]mcyst-LR | 967 | L | R | 0.8 |
| *Microcystis aeruginosa* PCC7806 | |  |  |  |  |
| 1 | [X, D-Asp^3^,DMAdda^5^]mcyst-LR | 967 | L | R | 0.1 |
| 2 | [D-Asp^3^, MeSer^7^]mcyst-LR | 999 | L | R | 0.1 |
| 3 | [X, DMAdda^5^]mcyst-LR | 981 | L | R | 0.0 |
| 4 | [X, DMAdda^5^]mcyst-LR | 981 | L | R | 0.1 |
| 5 | [X, D-Asp^3^,DMAdda^5^]mcyst-LR | 967 | L | R | 0.1 |
| 6 | [X, D-Asp^3^]mcyst-LR | 981 | L | R | 0.2 |
| 7 | [MeSer^7^]mcyst-LR | 1,013 | L | R | 0.4 |
| 8 | [D-Asp^3^]mcyst-LR | 981 | L | R | 52.2 |
| 9 | Mcyst-LR | 995 | L | R | 46.1 |
| 10 | [Dha^7^]mcyst-LR | 981 | L | R | 0.3 |
| 11 | [D-Asp^3^,Dha^7^]mcyst-LR | 967 | L | R | 0.4 |
| *Microcystis viridis* NIES102 | |  |  |  |  |
| 1 | [D-Asp^3^]mcyst-RR | 1,024 | R | R | 1.2 |
| 2 | Mcyst-RR | 1,038 | R | R | 9.7 |
| 3 | [Ser^1^, D-Asp^3^, Dha^7^]mcyst-LR | 983 | L | R | 0.1 |
| 4 | [D-Asp^3^]mcyst-YR | 1,031 | Y | R | 2.4 |
| 6 | [D-Asp^3^]mcyst-XR | 1,061 |  | R | 0.6 |
| 7 | Mcyst-YR | 1,045 | Y | R | 8.9 |
| 8 | [X, MeSer^7^]mcyst-(Hil)R | 1,027 | (Hil) | R | 0.1 |
| 9 | [D-Asp^3^]mcyst-LR | 981 | L | R | 2.5 |
| 10 | [Adda/DMAdda^5^]mcyst-L/HilR | 995 | L / Hil | R | 15.3 |
| 11 | Mcyst-HtyR | 1,059 | Hty | R | 1.7 |
| 12 | [X, D-Asp^3^]mcyst-HilR | 995 | Hil | R | 5.6 |
| 13 | Mcyst-HilR | 1,009 | Hil | R | 18.8 |
| 14 | Mcyst-FR | 1,029 | F | R | 2.0 |
| 15 | [D-Asp^3^]mcyst-WR | 1,054 | W | R | 1.5 |
| 16 | Mcyst-WR | 1,068 | W | R | 4.6 |
| 17 | [Dha^7^]mcyst-LR | 981 | L | R | 0.1 |
| 18 | [X, D-Asp^3^]mcyst-HilR | 995 | Hil | R | 0.2 |
| 19 | [D-Asp^3^]mcyst-XR | 1,021 |  | R | 0.1 |
| 5 | Mcyst-XR | 1,011 |  | R | 0.5 |
| 20 | Mcyst-XR | 1,059 |  | R | 0.1 |
| 21 | Mcyst-XR | 1,029 |  | R | 0.2 |
| 22 | Mcyst-XR | 997 |  | R | 0.7 |
| 23 | Mcyst-XR | 1,043 |  | R | 0.8 |
| 24 | Mcyst-XR | 985 |  | R | 0.2 |
| 25 | Mcyst-XR | 1,049 |  | R | 1.1 |
| 26 | Mcyst-XR | 999 |  | R | 0.3 |
| 27 | Mcyst-XR | 1,057 |  | R | 1.7 |
| 28 | Mcyst-XR | 1,025 |  | R | 0.5 |
| 29 | Mcyst-XR | 1,075 |  | R | 1.8 |
| 30 | Mcyst-XR | 1,069 |  | R | 0.2 |
| 31 | Mcyst-XR | 1,100 |  | R | 0.1 |
| 32 | Mcyst-XR | 1,077 |  | R | 0.3 |
| 33 | Mcyst-XR | 967 |  | R | 0.0 |
| 34 | Mcyst-XR | 1,086 |  | R | 0.1 |
| 35 | Mcyst-XR | 1,049 |  | R | 0.3 |
| 36 | Mcyst-XR | 1,091 |  | R | 0.7 |
| 37 | Mcyst-XR | 983 |  | R | 0.9 |
| 38 | Mcyst-XR | 1,063 |  | R | 0.9 |
| 39 | Mcyst-XR | 1,083 |  | R | 0.4 |
| 40 | Mcyst-XR | 1,040 |  | R | 0.3 |
| 41 | Mcyst-XR | 1,053 |  | R | 0.2 |
| 42 | Mcyst-XR | 1,023 |  | R | 0.4 |
| 43 | Mcyst-XR | 1,009 |  | R | 0.1 |
| 44 | Mcyst-XR | 1,037 |  | R | 0.5 |
| 45 | Unknown microcystin | 1,039 |  |  | 10.5 |
| 46 | Unknown microcystin | 969 |  |  | 0.3 |
| 47 | Unknown microcystin | 1,009 |  |  | 0.7 |
|  |  |  |  |  |  |
